# fMRI correlates of object-based attentional facilitation vs. suppression of irrelevant stimuli, dependent on global grouping and endogenous cueing

**DOI:** 10.3389/fnint.2014.00012

**Published:** 2014-02-10

**Authors:** Elliot D. Freeman, Emiliano Macaluso, Geraint Rees, Jon Driver

**Affiliations:** ^1^Cognitive Neuroscience Research Unit, Department of Psychology, City University LondonLondon, UK; ^2^Neuroimaging Laboratory, Fondazione Santa Lucia, I.R.C.C.S.Rome, Italy; ^3^Wellcome Trust Centre for Neuroimaging, University College LondonLondon, UK; ^4^Institute of Cognitive Neuroscience, University College LondonLondon, UK

**Keywords:** object-based attention, perceptual grouping, functional imaging, visual cortex, attentional modulation, coherent motion

## Abstract

Theories of object-based attention often make two assumptions: that attentional resources are facilitatory, and that they spread automatically within grouped objects. Consistent with this, ignored visual stimuli can be easier to process, or more distracting, when perceptually grouped with an attended target stimulus. But in past studies, the ignored stimuli often shared potentially relevant features or locations with the target. In this fMRI study, we measured the effects of attention and grouping on Blood Oxygenation Level Dependent (BOLD) responses in the human brain to entirely task-irrelevant events. Two checkerboards were displayed each in opposite hemifields, while participants responded to check-size changes in one pre-cued hemifield, which varied between blocks. Grouping (or segmentation) between hemifields was manipulated between blocks, using common (vs. distinct) motion cues. Task-irrelevant transient events were introduced by randomly changing the color of either checkerboard, attended or ignored, at unpredictable intervals. The above assumptions predict heightened BOLD signals for irrelevant events in attended vs. ignored hemifields for ungrouped contexts, but less such attentional modulation under grouping, due to automatic spreading of facilitation across hemifields. We found the opposite pattern, in primary visual cortex. For ungrouped stimuli, BOLD signals associated with task-irrelevant changes were lower, not higher, in the attended vs. ignored hemifield; furthermore, attentional modulation was not reduced but actually inverted under grouping, with higher signals for events in the attended vs. ignored hemifield. These results challenge two popular assumptions underlying object-based attention. We consider a broader biased-competition framework: task-irrelevant stimuli are suppressed according to how strongly they compete with task-relevant stimuli, with intensified competition when the irrelevant features or locations comprise the same object.

## Introduction

In our complex environment, with many different competing stimuli and goals, coherent behavior demands attentional selection. Such selection may both enhance relevant information and suppress irrelevant information. One important constraint on this selection is the perceptual organization of the stimulus into groups or proto-objects (Driver and Baylis, 1989; Palmer and Rock, 1994; Driver et al., 2001; Scholl et al., 2001). Classical studies (Kahneman and Henik, 1981; Baylis and Driver, 1993; Egly et al., 1994) show that it is easier to process, or harder to ignore visual stimuli when they are perceptually grouped with another stimulus at the current focus of attention. A popular explanation is that attention involuntarily spreads within the bounds of an object, automatically facilitating processing of all of its constituent parts and features (Duncan, 1984; Watson and Kramer, 1999; Driver et al., 2001; Vecera and Behrmann, 2001; Chen, 2012; but see Davis and Holmes, 2005). Such spreading may allow focusing activity on a specific object to the exclusion of others, for example eating off our own plate rather than our neighbors. But objects are commonly associated with other objects in a hierarchical structure (Baylis and Driver, 1993; Logan, 1996; Watson and Kramer, 1999), and sometimes we need to select just one individually, for example if we want to eat the peas on our plate but avoid carrots. If spreading of attention were always mandatory and facilitatory, we might find it difficult to “drill down” to specific sub-objects within a hierarchy, or their specific features, while ignoring others.

Some past research has examined how goal-driven attention or “perceptual set” (Neisser and Becklen, 1975; Vecera and Behrmann, 2001) might interact with grouping processes to parse the local constituents of a scene into task-relevant global structures (Baylis and Driver, 1993; Logan, 1996; Watson and Kramer, 1999; Freeman et al., 2001; Vecera and Behrmann, 2001; Khoe et al., 2006; Freeman and Driver, 2008). However the extent to which object-based allocation of attention is automatic or more intelligently prioritized continues to be debated (Chen and Cave, 2006, 2013; Richard et al., 2008; Yeari and Goldsmith, 2010; Shomstein, 2012; Zhao et al., 2013). Research on object-based facilitatory spreading of attention also often neglects the ‘dark side’ of visual attention (Tipper et al., 1991; Fuentes et al., 1998; Chun and Marois, 2002), namely suppression of irrelevant information. This paper examines the factors that may determine whether attentional selection facilitates or suppresses irrelevant features, or events at an irrelevant location of a scene, and how this may depend on the task-driven control of endogenous spatial attention, and stimulus cues for grouping.

Many past results seem consistent with the notion that excitatory attention spreads within the boundaries of a continuous object, but less readily across the gap between separate objects. For example, studies based on the popular cueing paradigm introduced by Posner et al. (1980) show that performance in discriminating a salient target on one end of a shape is improved if a previous cue has invalidly directed attention to the other end of the same shape, compared to when it cues the second shape (Egly et al., 1994). This is consistent with attentional spreading within the boundaries of the shape defining the “grouped array” (Avrahami, 1999; Vecera and Behrmann, 2001; Hollingworth et al., 2012). Other studies, using variants of the Eriksen flanker paradigm (Eriksen and Eriksen, 1974) show that stimuli seen as belonging to the same object tend to be processed automatically, leading to response competition (Kahneman and Henik, 1981; Driver and Baylis, 1989; Kramer and Jacobson, 1991; Baylis and Driver, 1992; Zhao et al., 2013). Several studies (Müller and Kleinschmidt, 2003; He et al., 2004; Martinez et al., 2006, 2007) found complementary effects in EEG and fMRI using similar stimuli and or cueing, for example showing an increased neural response to the unattended end of a rectangle when the other end was attended, relative to when another separate rectangle was cued.

A common claim in many of the above studies is that the grouped stimulus benefits from attentional spreading despite being irrelevant or even potentially disruptive to the task at hand. Such irrelevance is essential to the assumption that such spreading is based on automatic pre-attentive grouping processes (Vecera and Farah, 1994; Kramer et al., 1997; Weber et al., 1997; Davis et al., 2000; Vecera and Behrmann, 2001; Yeari and Goldsmith, 2010; Chen, 2012; Zhao et al., 2013). This claim may be challenged on two fronts. Firstly, past behavioral studies based on the Posner cueing paradigm have used cues that were intentionally unreliable, with the result that a task-relevant target could sometimes appear on uncued parts of the stimulus (i.e., in invalid-cue trials). In such situations of unreliable cueing, any evidence of apparent object-based attentional spreading might reflect a prior attentional set (or “attentional prioritization”) that preferentially includes grouped regions of the cued stimulus where targets are expected to appear, even if infrequently (Shomstein and Yantis, 2002; Müller and Kleinschmidt, 2003; Shomstein, 2012). Secondly, in paradigms based on the Erikson interference, the irrelevant flankers necessarily share potentially task-relevant properties with the target in order to provide measurable response interference. Such stimuli might attract attention due to their similarity (Harms and Bundesen, 1983; Baylis and Driver, 1992; Kim and Cave, 2001), perhaps via feature-based attention (Saenz et al., 2002; Martinez-Trujillo and Treue, 2004) or template-matching mechanisms (Duncan and Humphreys, 1989), while grouping processes might constrain such feature-based selection (Melcher et al., 2005; Festman and Braun, 2012), resulting in what appears as involuntary attentional facilitation. It therefore remains possible that under the right circumstances, facilitation might be eliminated or even become inhibitory if the probe stimuli are perfectly irrelevant and have nothing in common with the target stimulus.

Behavioral paradigms often suffer the limitation that any effect on “irrelevant” stimuli can only be assessed through overt responses, which may thus prime or direct attention to them. While some neurophysiological studies have just replicated the classic paradigms (e.g., Müller and Kleinschmidt, 2003; He et al., 2004), others have taken advantage of the possibility of measuring the brain's implicit responses to irrelevant stimuli. For example, Martinez et al. (2006, 2007) found enhanced evoked potentials for an irrelevant probe transient when participants were attending similar target transients on the opposite side of a rectangle figure. However, facilitation might still in principle have been caused by the activation of a “template” feature for detecting target transients. Another recent EEG study used frequency tagging to track flicker-evoked neural activity associated with a central target and a surround which either formed a continuous grating pattern with the center, or was segmented by a gap or phase offset (Kim and Verghese, 2012). Participants judged threshold increments in the contrast of the central component. This did not vary in the surround, which was thus entirely irrelevant. Surprisingly, activity to the surround was actually lower for the continuous pattern compared to discontinuous. This result would be consistent with suppression, rather than activation of wholly irrelevant grouped areas, perhaps as a result of more focal spatial attention (Kim and Verghese, 2012). However, the gap or phase manipulation may have introduced low-level features that modulated the amount of reciprocal center-surround inhibition, which in turn may have been amplified by a spread of attention across the surface. Manipulation of global rather than local grouping cues may be preferable to avoid this ambiguity.

While the above studies typically manipulated grouping between patterns occupying different regions of space, other studies have attempted to control spatial attention to obtain a purer measure of the capabilities of object-based attention in selecting one group in the presence of a second overlapping pattern (e.g., Duncan, 1984). Many studies manipulated grouping via spatio-temporal cues such as common-fate motion (e.g., O'Craven et al., 1999; Jarmasz et al., 2005), for example using transparent coherently-moving dots (Valdes-Sosa et al., 1998; Schoenfeld et al., 2003; Mitchell et al., 2004; Ciaramitaro et al., 2011). Here selection of one feature tends to activate selection of other features that are bound to the same object, even if they are too faint to be consciously perceived (Melcher and Vidnyánszky, 2006), and may strongly suppress physiological responses to the irrelevant object (Valdes-Sosa et al., 1998). Again it is often claimed from this that the spread of attention is inevitable, and facilitatory to all features belonging to the object that is attended. In support of this a recent fMRI study found selective enhancement of frequency-tagged irrelevant features belonging to a relevant dot pattern, in context of irrelevant overlapping dot pattern (Ernst et al., 2013). However, a behavioral study found that cueing of a feature results in selective speeding of responses to it, but did not facilitate responses to other irrelevant features belonging to the same object (Wegener et al., 2008). One critical difference between this study and others, as Wegener et al. (2008) suggest, may be that the target stimulus did not overlap with a distractor stimulus (see also Davis and Holmes, 2005 for consideration of further stimulus factors which may determine within-object benefits vs. costs). In cases of overlap (e.g., Valdes-Sosa et al., 1998; O'Craven et al., 1999; Schoenfeld et al., 2003; Mitchell et al., 2004; Jarmasz et al., 2005; Ciaramitaro et al., 2011; Ernst et al., 2013), we might accrue evidence from any available redundant cues, such as a contrasting color or sudden change in motion trajectory of the targets dots, that might help to uniquely distinguish the target features from features belonging to an overlapping distractor, even if they are not consciously detected (cf. Melcher and Vidnyánszky, 2006). Such additional cues are of less relevance when there are no confusable features within the spotlight of spatial attention, and might thus be ignored more effectively when they are irrelevant. Thus, while object-based spreading of attention may appear mandatory in the above studies using overlapping stimuli, there might be less attentional spreading if the stimuli did not need to be segmented in order to be selectively attended.

It might be concluded from this previous work that facilitation across features within an object is not mandatory but dependent on the need to segment a target from competing overlapping or surrounding features when present. However it is not yet clear whether object-based attentional allocation to completely irrelevant stimuli is also optional across space to similar features of different stimuli, whether it is always facilitatory, and how this might depend on global (rather than local) grouping cues. To address these issues, we used fMRI to measure neural activity associated with any interactions between global grouping (by common-fate motion) between two stimuli in opposite visual hemifields. We measured the effect of attending to subtle targets in one vs. the other hemifield, while also independently measuring BOLD signals evoked by highly salient but completely irrelevant transient color changes within the attended or ignored hemifield. We focused our analyses on relevant areas of motion-sensitive or retinotopic early visual cortices (see Methods) and assessed whether effects of attention and grouping mostly affected feature-specific activity in color and motion related areas (i.e., V4 and V5/MT respectively), and/or whether there were more general effects on early visual cortices (e.g., see Ciaramitaro et al., 2011). If object-based attentional spreading is generally mandatory and facilitatory both within and between objects, irrelevant transients should always evoke an increase in signals associated with attended stimuli, and also in the opposite hemifield specifically when it is grouped with the attended hemifield. More generally, attention-related activations (associated with the blocked manipulation of hemifield cueing) should leak over to the unattended hemifield under grouping. The contrasting hypothesis is that attentional spreading may be less facilitatory or even inhibitory toward features that are entirely irrelevant to the task and of no use for image segmentation.

## Methods

### Participants

Eight participants aged between 25 and 35 participated with their informed consent. All had normal or corrected-to-normal vision, and had previous experience of imaging experiments, but were naïve to the purpose of the present study. The experiment was approved by the local ethics committee.

### Stimuli

**Figure 1:**
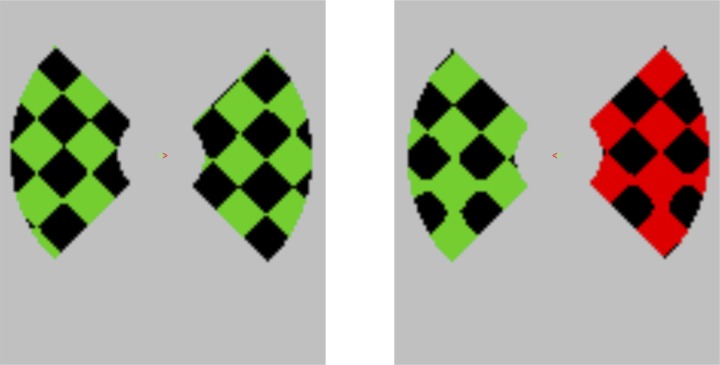
**Sample stimulus displays. Left panel:** No transients, no targets; central red arrow is cueing rightwards attention. **Right panel:** Transient color change on right hemifield, and a target check-size change is also shown in both hemifields in the lower quadrant. Upper vs. lower location was random and independent for each hemifield, and participants had to indicate the location of the target in the cued hemifield (here on the bottom left).

An LCD projector back-projected stimuli onto a screen at the rear of the magnet bore. Video mode was 640 × 480 with screen refresh rate of 60 Hz, and output was linearized using 8-bit software gamma-transformation. Observers lay supine in the scanner, and viewed the screen via a mirror mounted on the head coil, across a total viewing distance of 62 cm. Stimulus presentation and timing was controlled by a PC running MATLAB (Mathworks Inc.) and COGENT 2000 toolbox (http://www.vislab.ucl.ac.uk/Cogent2000/). The visible display subtended visual angles of 31° horizontal by 14° vertical. Displays were composed of two light and dark diagonally oriented checkerboards (each composed of the product of two orthogonal oblique sinusoidal gratings of wavelength 4.84°, thresholded with light coloring for positive values and black for negative). This resulted in diamond-shaped checks with edges measuring 2.40° in length. Checkerboards were presented on a mid-gray screen on each side of the vertical midline, visible through 90° segments of a central annulus-shaped sharp-edged window, with inner and outer diameter of 2.82° and 9.8° respectively (see Figure 1). Each checkerboard translated behind the window along a circular path of radius 1.5°, taking 2 s to complete each cycle. Grouping was manipulated by moving left and right hemifields either in phase with each other or 90° out of phase. In-phase motion produced the impression of a continuous checkerboard surface passing behind left and right apertures (see Movie 1); out-of-phase motion gave the appearance of two independent checkerboards (Movie 2).

Fixation stimuli consisted of small “<” and “>” characters at the center of the visible display, one red and the other green interchangeably. On every 2 s motion cycle, synchronously but independently on both sides, the light checks on either the upper or lower quadrant smoothly expanded in size by a maximum of 0.2°, while the dark checks contracted in size by the same amount, with maximum size change at mid-cycle, returning again to their original size at the end of the cycle. This was achieved by adding a 2D Gaussian function to the combined oblique gratings composing the chequerboard (*SD* 1.2°, positioned ±1.7° horizontally and ±0.68° vertically relative to the fixation point), prior to thresholding, and modulating the amplitude of this function with a Gaussian temporal profile (*SD* 0.33 s). Participants were required to discriminate between upper and lower check-size changes on the hemifield pointed to by the red fixation arrow, while ignoring all changes on the opposite hemifield (which were anyway uninformative). Participants made “up” or “down” responses using one of two keys on an optical fiber button-box. The importance of responding on every motion-cycle “trial” was emphasized. Eye position data were sampled at a frequency of 60 Hz during scanning using remote-optics infrared eye tracking (ASL 504, Applied Science Laboratories, Bedford, MA). The importance of maintaining fixation on the central arrow stimuli was emphasized to participants.

For most of the time, the checkerboards were colored green on a black background, but would occasionally flash red on one hemifield or the other for a duration of 500 ms. These events were not temporally correlated with the check-size changes. Subjective red-green isoluminance was established for each participant prior to scanning, using method of adjustment to minimize perceived flicker of a 30 Hz alternating red-green checkerboard.

### Design and procedure

Participants first attended a half-hour training session in a psychophysics laboratory. They were familiarized with the task and were given verbal feedback on their eye-movements during the task. The maximum check-size change determined task difficulty, and a method of constant stimuli was used to find the 85% accuracy level for each participant, which was used throughout the scan. In the scanner, participants first completed the isoluminance adjustments and eye-tracker calibration. There then followed 10 four-minute scans. Each of these runs was divided into four one-minute blocks, presented in counterbalanced order.

Each block represented a different crossing of two independent variables: Grouping (in-phase or “Grouped” motion vs. out-of-phase or “Ungrouped” motion), and Attention (to left vs. right hemifields. This 2 × 2 block design was superimposed on an event-related design, in which transient red flashes occurred independently on left and right hemifields, every 2–12 s for a period of 500 ms (e.g., see right of Figure 1). In each block, five flashes would occur on each hemifield, independently and unpredictably. A given flash, therefore, could be classified as occurring on an attended side or an unattended side, and on a checkerboard that was either grouped or segmented from its opposite counterpart.

### Neuroimaging

BOLD contrast fMRI images were acquired in a Siemens Allegra 3 Tesla MRI scanner (Siemens, Erlangen, Germany), using an EPI sequence. Slices were positioned to cover the whole brain. Voxel size was 2 × 2 × 2 mm. There were 10 scanning runs for each participant, each lasting 4 min 40 s, and consisting of 85 volumes sampled with repetition time of 3.12 s and 48 slices per volume. Volumes had 48 slices of 2 mm thickness with a 1 mm gap between slices, giving a resolution of 3 × 3 × 3 mm. We also acquired T1-weighted MPRAGE images for structural analysis with a resolution of 1 × 1 × 1 mm.

### Localization

Retinotopic visual areas (i.e., V1, V2, V3, V3A, and V4) were each identified on the basis of standard rotating-wedge scans conducted in a prior session, with segmentation and cortical flattening using MrGray software (Teo et al., 1997; Wandell et al., 2000). These retinotopic regions of interest (ROIs) were then inclusively masked by *t*-maps representing voxels that were significantly activated (at *p* < 0.05 uncorrected) by the stimuli across all blocked conditions. We identified regions of interest corresponding putatively to motion-sensitive areas, for individual participants based on voxels showing significant activation (*p* < 0.001 uncorrected) across all blocked conditions, whose coordinates were consistent with the published location of area hMT/V5+ (e.g., Watson et al., 1993; Tootell et al., 1995; Hasnain et al., 1998). As the above localization analyses were based on BOLD signal averaged across all block-related conditions (attention left vs. right, and grouped vs. ungrouped) this method of localization could not bias the outcome of our tests for hypothesized differences between conditions, either block-related or event-related. This method of defining a region of interest, based on a contrast that is orthogonal to those used to test an experimental hypothesis, is an established approach in the literature (Friston et al., 2006). We used our own circularly translating stimuli, rather than a traditional independent motion localizer based on moving random-dot kinematograms, as this could isolate regions sensitive to the specific type of motion used in our main experiment, providing a principled and statistically independent way to identifying relevant voxels that might be subject to our particular modulations of spatial attention and stimulus grouping. However, given that these areas were not identified using standard functional localizers, the label “hMT/V5+” is used tentatively.

### Eye-movement control

**Figure 2:**
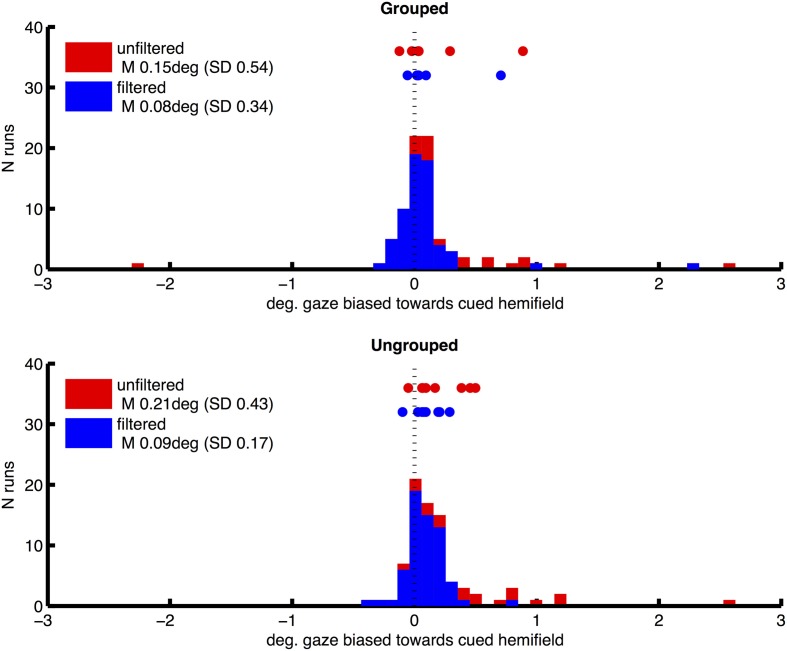
**Distribution of horizontal gaze locations relative to central fixation (0), toward the cued hemifield (positive values) or away (negative), in degrees of visual angle**. Height of bars indicates the number of scanning runs associated with each gaze value. Distributions are shown separately for grouped and ungrouped conditions (upper and lower graphs respectively). Red and blue coloring depicts unfiltered and filtered eye-data respectively (see Methods for details). Datapoints shown above the distributions mark mean horizontal gaze locations for individual subjects.

Prior to fMRI analysis, we used eye position data from eye tracking to control for the possibility that attentional cueing to left and right hemifields, and our manipulation of grouping, could systematically affect participants' fixation patterns. For each run, eye-tracker data (X and Y coordinates for each 16.6 ms acquisition frame) were processed to remove any linear trend, and filtered to exclude blinks or signal drop-outs. Frames in which there was a horizontal deviation from central fixation of greater than 2° were then identified, which might be caused if participants made saccades toward one of the hemifield stimuli. From these we derived a measure of fixation bias toward the attended hemifield, for each scanning run in each participant, by subtracting the proportion of fixation deviations away from the cued hemifield, from the proportion of deviations toward the cued hemifield. This bias measure was therefore positive when participants made more saccades of greater than 2° horizontally toward the cued hemifield. The distribution of this measure over runs and participants had a long tail toward higher values (mean 0.0084, *SD* 0.188, skewness 3.63), consistent with the occasional tendency to peek at the hemifields containing the task-relevant targets. We attempted to correct this by excluding individual runs in which this fixation bias measure had values of greater than 0.01. This resulted in omission of 23% of runs on average across participants (*SD* 18%), and a more symmetrical distribution of gaze bias scores (mean 0.0017, *SD* 0.0048, skewness −1.77) and mean gaze locations (see Figure 2, plotting frequency of horizontal gaze locations toward vs. away from the cued hemifield, before and after the above correction for bias, for the two grouping conditions separately). Following this correction for bias, we compared the effects of left/right cueing and grouping on mean horizontal gaze coordinates based on ten scanning runs per subjects, in a two-way repeated measures ANOVA. Results showed no significant bias toward the cued hemifield [*F*_(1, 7)_ = 3.32, *p* = 0.11, no main effect of grouping *F*_(1, 7)_ = 0.43, ns] and no significant interaction [*F*_(1, 7)_ = 0.002, ns].

### fMRI analysis

Preprocessing and analysis of fMRI images was conducted using SPM2 (http://www.fil.ion.ucl.ac.uk/spm). The first five images of each scanning run were discarded to allow for magnetic saturation effects. The remaining images were realigned and coregistered to the individual participants' structural scans for analysis of early retinotopic areas. A high-pass filter was applied at 0.0078 Hz to remove low-frequency signal drifts. For whole-brain analysis, images were spatially normalized into standard space (MNI) and spatially smoothed with a Gaussian kernel of 8 mm FWHM.

For each participant, data were entered into a general linear model (Friston et al., 1994) specifying blocked variables and transient events as separate regressors convolved with a canonical HRF, within the same model. Each model therefore had four regressors for each of the four block types in the 2 × 2 (attention × grouping) design, in addition to eight further event-related regressors corresponding to the four conditions of the 2 × 2 design for the left and right transient events independently. Whole-brain random-effect analyses were then performed using one-sample *t*-tests, to assess the statistical significance of selected contrasts across participants. Block-related contrasts compared BOLD signals for left vs. right attention, and grouped vs. ungrouped stimuli. Event-related contrasts compared left vs. right transients, in the context of grouped vs. ungrouped stimuli. We also tested contrasts which assessed the hypotheses that the difference between left and right blocked attention, or signal evoked by left vs. right transients, was greater (or smaller) under grouped vs. ungrouped conditions.

## Results

### Behavioral data

One participant failed to respond on 16% of trials, compared to an average failure rate across the remaining participants of only 0.75% (*SD* 0.5%). Proportion correct was calculated for up-down discrimination of check-size changes after filtering out missed trials. Mean accuracy across all participants was 91% (*SD* 3%). The same participant with high miss-rates also had the poorest accuracy (87%). After excluding this participant, the filtered accuracy data were analyzed in a repeated-measures ANOVA, with attended hemifield (Left vs. Right) and Grouping (Grouped vs. Ungrouped stimuli) as repeated-measures factors. There was a significant main effect only for Attention, with higher accuracy for discriminating check-size changes in the left hemifield [*F*_(1, 7)_ = 9.75, *p* = 0.02]. Mean (and *SD*) for left was 94% (0.3%) and for right, 91% (1%). There was no significant interaction with grouping. A similar pattern was observed with the full data set.

### Whole brain analyses

**Figure 3:**
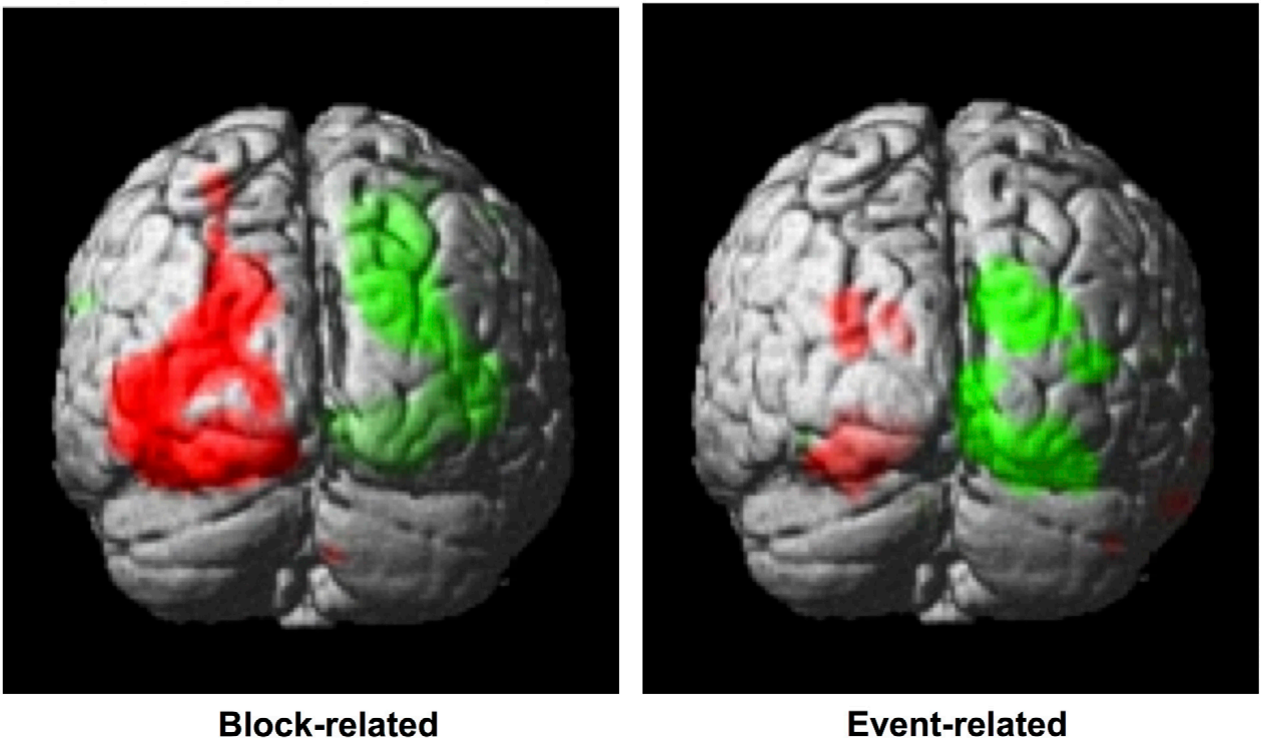
**Whole-brain analyses. Left:** Block-related contrast of left vs. right-cued conditions, under similar stimulus conditions (highlighted in green and red, respectively). These attention-driven areas were used to mask visual areas distinguished using retinotopy, to define our regions of interest. **Right:** Event-related contrast of left vs. right color-change transients (green and red, respectively). Results are superimposed on a standard template, with a threshold of *p* < 0.001 uncorrected.

Statistical contrasts of left vs. right cued attention revealed significant activations (family-wise corrected for multiple comparisons at *p* < 0.05) contralateral to the cued hemifield in cuneus and lingual gyrus (see Table 1 for coordinates). Lowered thresholds (*p* < 0.001 uncorrected, see Figure 3, left) revealed widespread activations only in posterior visual areas contralateral to the cued hemifield. Event-related contrasts of left vs. right transients showed significant activations contralateral to the transient (corrected for multiple comparisons at *p* < 0.05) in lingual gyrus and fusiform gyrus (Table 1). At lower thresholds (e.g., *p* < 0.001 uncorrected, Figure 3, right) activations were seen in occipital inferior and superior occipital areas putatively within area V3a (Tootell et al., 1995). Contrasts of grouped vs. ungrouped stimuli revealed no notable activations even at lowered thresholds (*p* < 0.001 uncorrected), for either event-related or block-related analyses. There were also no significant results for whole-brain block and event-related analyses of specific interactions between grouping and attention.

**Table 1:** **MNI coordinates (mm) of regions identified in whole-brain contrasts, significant at *p* < 0.05 corrected**.

**Contrast**	**Area**	***x*^1^**	***y***	***z***	***T***
**BLOCK-RELATED**					
L–R	Cuneus	24	−94	20	6.72
R–L	Cuneus	−26	−92	29	8.22
	Lingual gyrus	−26	−80	−10	5.19
**EVENT-RELATED**					
L–R	Fusiform gyrus	32	−64	−18	7.12
R–L	Lingual gyrus	−26	−84	−12	4.31

1 *MNI coordinates (mm)*.

### Retinotopy: blocked analyses

Beta weights (representing the overall level of BOLD activation) for each of the block-related conditions were estimated from each of the ROI's for each participant (i.e., masked retinotopic visual areas including hMT/V5+, see Methods). As we had no specific hypotheses about the laterality of attentional effects, data from left and right hemispheres were pooled according to whether the respective contralateral visual hemifield was attended or ignored. Data were entered into a 3-way repeated measures ANOVA with the following factors: Attention (whether a given ROI was contralateral vs. ipsilateral to the attended hemifield), Grouping, and cortical Area.

Initial analysis revealed a main effect of Attention, with larger BOLD signals when attention was cued to the contralateral vs. ipsilateral hemifield [*F*_(1, 7)_ = 70.69, *p* = 0.0001]; and a main effect of Grouping, with greater signals for ungrouped than grouped stimuli [*F*_(1, 6)_ = 9.29, *p* = 0.019]. There was a main effect of Area [*F*_(5, 35)_ = 23.86, *p* < 0.0001], which was partially accounted for by higher signal estimates in hMT/V5+ (9.87, *SD* 0.14) compared to the other areas (mean 4.86, *SD* 0.87). Variability was also much higher in MT/V5+ (*SD* 0.14) compared to other areas (mean *SD* 0.022, *SD* 0.017). The only significant interaction was between Attention and Area [*F*_(5, 35)_ = 4.11, *p* = 0.005], with larger effects of attention to the contralateral vs. ipsilateral hemifield in hMT/V5+ than in the other visual areas (see Supplementary Figure 1).

**Figure 4:**
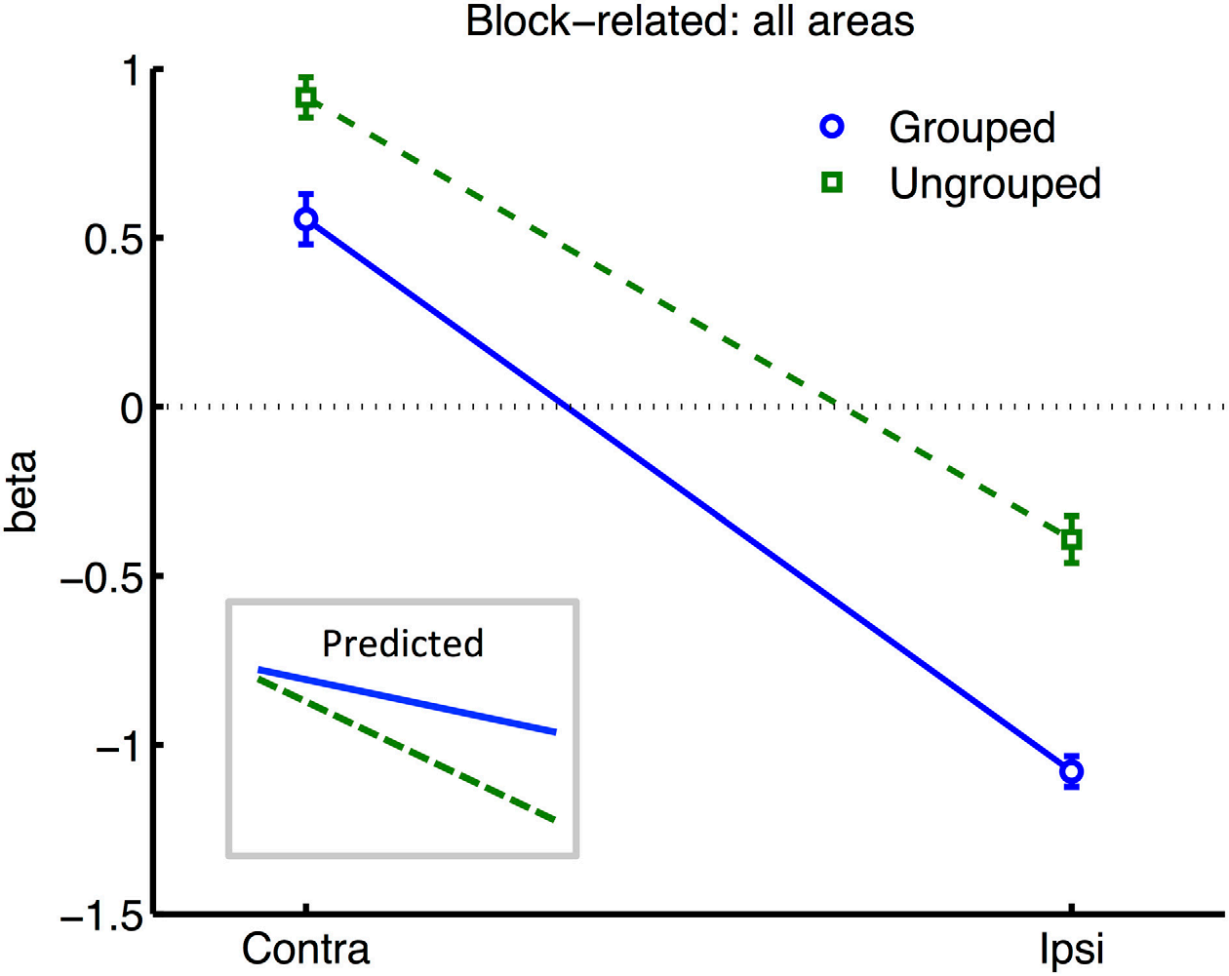
**Results from retinotopic analysis of Block-related BOLD, averaged across all visual areas**. Normalized (see Methods) beta weights are plotted for hemispheres contralateral vs. ipsilateral to the cued visual hemifield, for grouped (blue circles and solid lines) vs. ungrouped stimuli (green squares and dashed lines). In all graphs, error-bars indicate one unit of within-subjects standard error. Inset shows predictions assuming facilitatory and automatic spreading of object-based attention.

All further analyses excluded the participant with poor hit rates, in case this was indicative of poorly controlled attention. To render the variances between all ROI areas more uniform, we normalized each participant's beta estimates for each given condition from each given ROI (pooling data across hemispheres, as described above), by subtracting the average (and dividing by the standard deviation) of block-related beta estimates obtained across all conditions from the same bilateral ROIs. Normalized data now had similar means and ranges across ROIs and participants, while the detailed pattern of results across conditions remained unchanged within each ROI. An ANOVA based on the normalized data now revealed a significant interaction of Attention and Grouping [*F*_(1, 6)_ = 8.06, *p* = 0.03], with greater attentional modulation in the grouped compared to ungrouped condition (see Figure 4). These analyses also confirmed the main effect of Attention [*F*_(1, 6)_ = 705, *p* < 0.0001] and Grouping [*F*_(1, 6)_ = 22.12, *p* = 0.003], and no main effect or interaction for Area.

**Figure 5:**
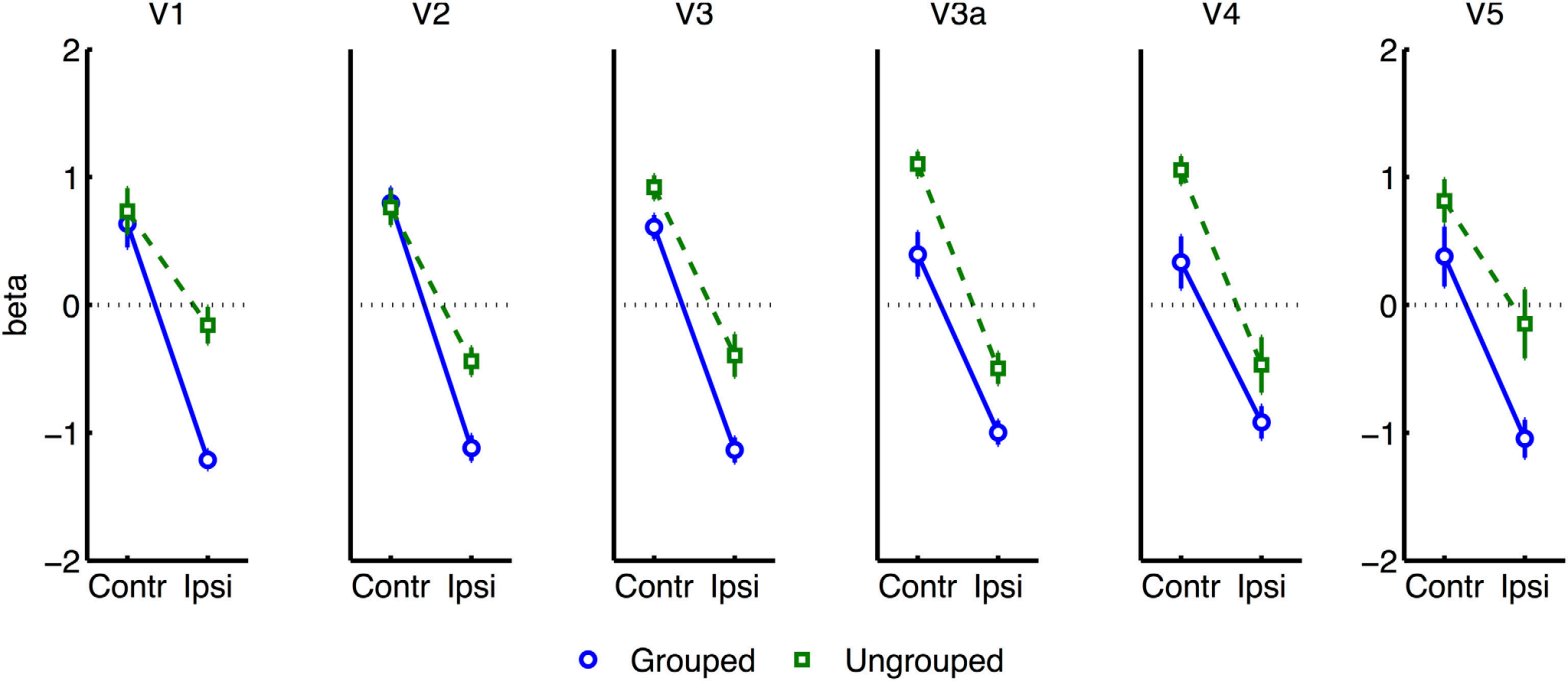
**Normalized block-related results for each area of visual cortex**.

Separate analyses were conducted for each visual area showed that the interaction between grouping and attention was significant in V2 [*F*_(1, 6)_ = 13.62, *p* = 0.01], and borderline significant in V1 [*F*_(1, 6)_ = 5.40, *p* = 0.059] (see Figure 5). A similar pattern was observed for the non-normalized data [V1: *F*_(1, 6)_ = 5.18, *p* = 0.063; V2: *F*_(1, 6)_ = 9.05, *p* = 0.023] (Supplementary Figure 1). Including the participant with high miss-rates rendered these effects non-significant.

### Event-related analyses

In the event-related analyses, beta weights were estimated from each ROI for each participant. As we had no specific hypotheses about the left vs. right location of the transient stimuli, transient events were coded according to whether they appeared in the attended vs. ignored hemifields, and whether they appeared in the hemifield contralateral or ipsilateral to the ROI. A four-way ANOVA included these two factors (Attention and Transient Location) along with Grouping and cortical Area.

Initial analysis of the whole sample (excluding the participant with poor hit rates) revealed only the main effect of contralateral vs. ipsilateral Transient Location as significant. Examination of the raw data revealed one participant with highly disparate beta estimates in particular ROIs and conditions (contributing to an increased range of betas ±15 compared to ±5 for other participants, and increased standard deviation of 3.5, while others varied between 1 and 2.3, resulting in a *z*-score of 2.13 with respect to the whole sample).

Similar to the block-related analysis, we normalized the data for each ROI to render the variances between participants and areas more uniform. This was done for each participant, and for each pair of bilateral ROIs, by taking the beta estimates obtained for each condition, subtracting the average beta across all conditions obtained from the same bilateral ROIs (pooling data across hemispheres, as before), and then dividing by the standard deviation of that same sample. All participants and ROIs now had data varying across a similar range, while the fine pattern of results across conditions within ROIs was not affected. ANOVA based on these data showed a significant main effect of Transient Location, where contralateral transients produced greater activation than ipsilateral transients [*F*_(1, 6)_ = 82.44, *p* = 0.0001]. There was a significant interaction between Cortical Area and Transient Location, with apparently less difference between the response to ipsilateral and contralateral events in V5 relative to other areas [*F*_(5, 30)_ = 4.60, *p* = 0.003]. The only other significant interaction was between Area, Grouping and Attention [*F*_(5, 30)_ = 2.77, *p* = 0.036].

**Figure 6:**
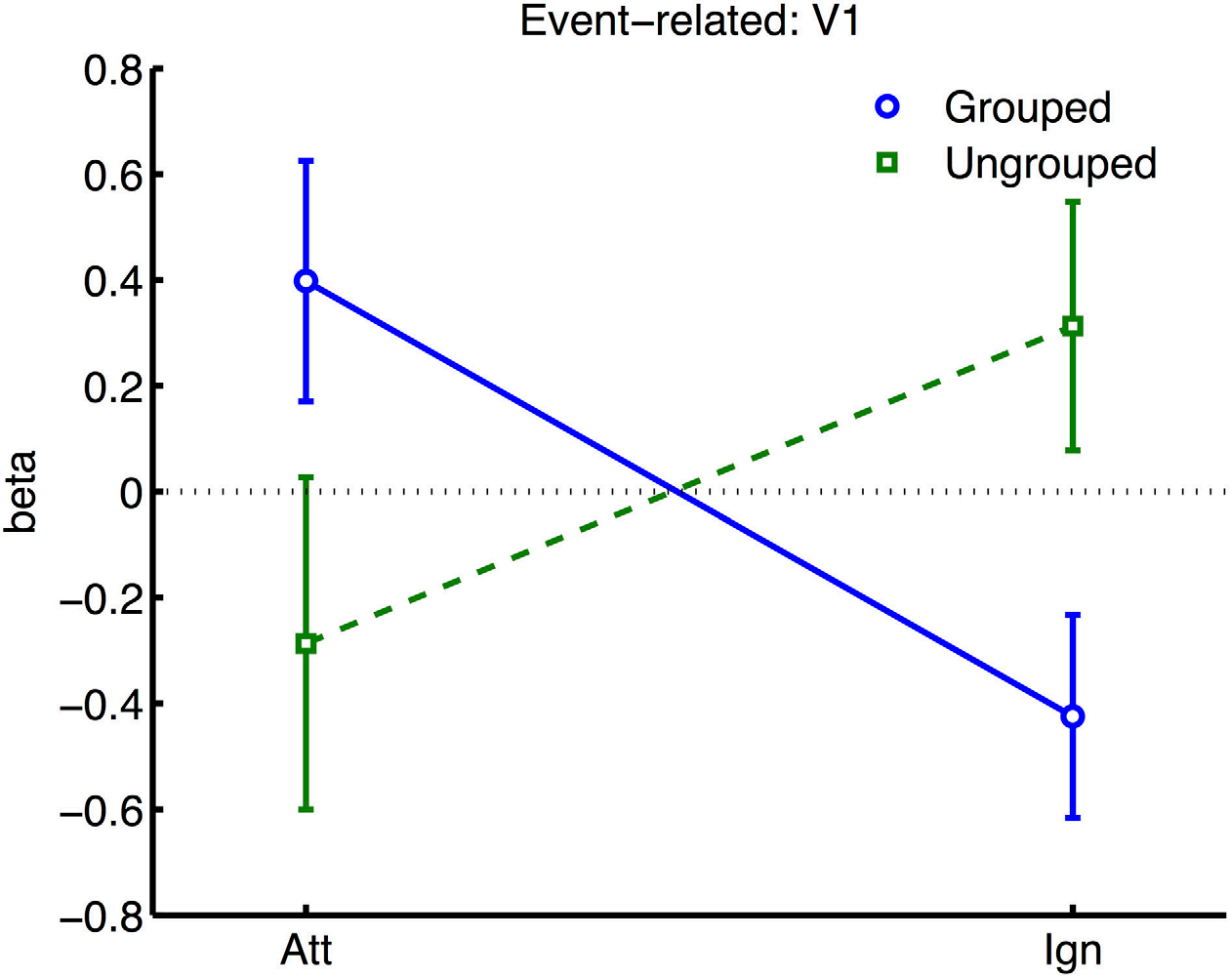
**Results from retinotopic analysis of Event-related BOLD, showing the interaction between grouping and attention, which was significant in area V1 only**. Normalized beta-weights (see Methods) are plotted as a function of whether the color-change transients were on the attended or the ignored hemifield, for grouped (blue circles) vs. ungrouped stimuli (green squares). Results are averaged across areas contralateral and ipsilateral to the location of the transient.

To further explore this latter interaction we analyzed normalized results for each visual area (see Figure 6), in separate three-factor ANOVA's for attention, grouping, and contralateral vs. ipsilateral hemifield (excluding the participant with high miss-rates). The main effect of contralateral vs. ipsilateral hemifield was significant in all areas except V5 [V5: *F*_(1, 6)_ = 0.29, ns; other areas: *F*_(1, 6)_ ≥ 43.80, *p* ≤ 0.0006]. The interaction between Grouping and Attention was significant in V1 only [*F*_(1, 5)_ = 9.51, *p* = 0.022]. As shown in Figure 6, this interaction took the form of a cross-over interaction: under grouped conditions, transients evoked stronger BOLD signals when occurring within the attended compared to the unattended hemifield; conversely in the ungrouped condition, transient events evoked more response in the unattended compared to the attended hemifield. To take a specific example, when participants attended left, a left transient was associated with a stronger response than a right transient, in the grouped context, but a weaker response compared to a right transient in the ungrouped context (with the complementary situation occurring for right transients under attention to the right hemifield).

**Figure 7:**
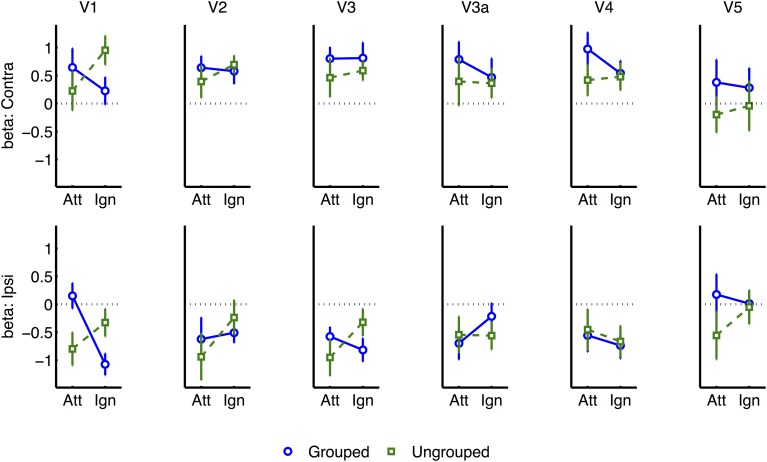
**Event-related normalized results for each visual region of interest, and for contralateral vs. ipsilateral transients**.

Similar but non-significant trends [*F* ≤ 1.38] for the interaction between Attention and Grouping were apparent in areas V2, V3, and V4 (Figure 7). There were no other significant effects, and in particular no significant trends from the two-way or three-way interactions, to indicate that the spread of activations between contralateral and ipsilateral ROIs was significantly dependent on grouping [all *p* ≥ 0.1].

A similar pattern of results and in particular the significant V1 interaction between Grouping and Attention were also observed in the non-normalized data [*F*_(1, 5)_ = 7.82, *p* = 0.038] (Supplementary Figure 2), after excluding the participant with unusual variance.

## General discussion

To test the predictions of object-based attention theories, here we measured the BOLD signals evoked by a completely irrelevant transient color change, as a function of grouping between attended and to-be-ignored hemifields. We observed a cross-over interaction between grouping and attention-related effects on BOLD signals evoked by the task-irrelevant transients, which was significant in retinotopic area V1. When both hemifields were grouped by common-fate motion, the transient stimulus evoked greater activation when it appeared in the attended hemifield compared to unattended. Conversely, when each hemifield moved independently, the transient evoked greater activity when it appeared in the ignored hemifield compared to attended. As explained below, our findings challenge the assumptions often made in studies of object-based attention, that attentional spreading within and between objects is usually automatic and facilitatory.

Many studies of object-based attention have concurred that spatial attentional influences spread automatically between parts of a display which are grouped, and that the effect of this spreading is largely a facilitation of the processing of these parts (Driver and Baylis, 1989; Kramer and Jacobson, 1991; Egly et al., 1994; Müller and Kleinschmidt, 2003; Martinez et al., 2006, 2007; Hollingworth et al., 2012; Zhao et al., 2013). If facilitation were generally the case, we should have observed an increase in activation evoked by transients in the unattended hemifield specifically when this hemifield was grouped with the attended hemifield, compared to when it was moving independently (see inset in Figure 4 for the predicted pattern). We found the opposite: a decrease in activation under grouping compared to no grouping (right datapoints of Figure 6). Furthermore, if object-based spreading were always automatic, there should have been no effect of manipulating attention to different parts of a grouped display, yet V1 showed a clear benefit for transients which were part of the attended vs. ignored hemifield (blue circles in Figure 6).

Our results further challenge the common assumption that facilitatory attentional influences spread automatically between features bound *within* a single object (Duncan, 1984; Valdes-Sosa et al., 1998; O'Craven et al., 1999; Schoenfeld et al., 2003; Mitchell et al., 2004; Jarmasz et al., 2005; Ciaramitaro et al., 2011; Ernst et al., 2013). If always facilitatory, no specific difference in the response to transients due to grouping should have been expected for stimuli currently under the focus of attention, as the grouping manipulation should not have affected local binding of target and transient features. However, in our results, facilitation of irrelevant within-object features was observed only when the stimulus was grouped with the opposite hemifield, with eliminated facilitation when the attended stimulus was segmented from its counterpart by motion cues (compare left datapoints of Figure 6). Furthermore, if spreading were always automatic, it should also not depend on the allocation of spatial attention to different parts of a group, but here we observed that facilitation to transients increased under spatial attention with grouped stimuli (and decreased under spatial attention, without grouping). We next consider the factors that might underlie this pattern of results.

### Comparison with previous studies

Our study has much in common methodologically with previous studies discussed above, which generally required an attentionally demanding discrimination of subtle features belonging to one object, while ignoring other features belonging to the same and/or different objects. For example, we used a spatial discrimination of check-size modulations in upper vs. lower quadrants of the cued hemifield, to encourage subjects to spread their attention over the whole hemifield stimulus rather than focusing exclusively on one location. This is analogous to paradigms based on Egly et al. (1994) in which subjects must discriminate an event such as a luminance increment, that can occur unpredictably on one or other end of a stimulus shape. Note that the luminance modulations defining the target and distractor check-size events in our experiment could not have confounded our event-related measures of the response to transient color-flash events, which were presented bilaterally at temporally uncorrelated periods during the trial, rather than synchronized with the check-size changes.

One critical difference with previous work is that here we measured the effects of attentional spreading to completely irrelevant transients. In our paradigm, the transient red flashes were entirely irrelevant to the task of discriminating subtle upper vs. lower quadrant check-size changes, and contained no features that were confusable with the target. This contrasts with many of the above studies, in which the stimulus to which attention spreads is either not entirely irrelevant, or shares some common features with the relevant target. This methodological difference might help to explain why the present results did not show any evidence of facilitatory attentional spreading between grouped stimulus parts, but rather indicated a reduction of facilitation (or suppression) under specific attention and grouping conditions.

Another methodological contrast is that previous studies indicating automatic and facilitatory spreading of attentional resources between features within an attended object have often presented target stimuli transparently overlapping with distractor stimuli (Valdes-Sosa et al., 1998; O'Craven et al., 1999; Melcher et al., 2005; Ernst et al., 2013), thus creating a segmentation problem. To resolve this problem, one strategy might be to recruit information from other (nominally irrelevant) features of the target stimulus that are uniquely associated with the target (Wegener et al., 2008). Here, our use of opaque stimuli removes this particular segmentation problem, at least in the ungrouped condition. This could account for the lack of facilitatory spreading between relevant and irrelevant features within the attended hemifield, observed in the ungrouped condition in the form of a reduced response to transients in the attended hemifield (e.g., see left datapoints of Figure 6). However in the grouped condition, an analogous segmentation problem might arise, where the target hemifield must be distinguished from the opposite hemifield with which it is grouped. In this case selective attention to the target in one hemifield may be aided by spreading facilitatory attention to the transient cues, which help to define the context of the hemifield in which the target appears. Such attentional facilitation of irrelevant features belonging to the target may thus help to resolve the segmentation problem and improve selection of the task-relevant features, and could explain the apparent attentional spreading to transients within the attended hemifield, specifically under grouping (Figure 6).

The need for segmentation might also explain the apparently similar findings of Kim and Verghese (2012), who reported a reduction rather than an increase of the physiological response to a completely irrelevant surround stimulus in the presence of a grouped vs. segmented central target stimulus. They proposed that the demanding central contrast detection task required a withdrawal of spatial attention from the irrelevant surround specifically under conditions of grouping, where presumably the surround becomes more distracting. A similar account might also explain the present block-related results (Figure 4), showing specifically decreased BOLD signal in the unattended hemifields under grouping compared to no grouping. However an alternative account of Kim and Verghese's (2012) result is that attention modulated inhibitory lateral interactions between the closely abutting center and surround stimulus. In common with some other studies manipulating local stimulus features (e.g., Egly et al., 1994; Murray et al., 2002; Altmann et al., 2003; Martinez et al., 2006, 2007), this creates a potential ambiguity over whether top-down or local (“horizontal”) interactions between stimulus parts may be the neural substrate of attentional spreading. Local spreading might function via a mechanism of ‘incremental grouping’ (Roelfsema, 2006) whereby signals are transmitted between visual areas along object boundaries via excitatory horizontal lateral interactions (Avrahami, 1999). These lateral interactions may themselves be gated by attention (Freeman et al., 2001; De Meyer and Spratling, 2009). Such local interactions were controlled in the present study because hemifields were always separated by a gap (2.66°), while the local structure and motion of each hemifield was identical under both grouping conditions. By manipulating only the spatio-temporal relationship between the hemifields, any resulting differences in BOLD response in early visual cortex may be more readily attributable to top-down grouping mechanisms associated with differences in perceived grouping between hemifields, rather than the local structure of visual stimulation presented within each hemifield.

An account based on top-down influences receives further support from our observed grouping-by-attention interaction even within visual areas ipsilateral to the transient stimulus. This effect was independent from a significant effect of contra>ipsilateral areas for transient events. Thus while the stimulus-driven response to transients remained strongly localized to contralateral areas, this was observed in the context of general increases or decreases of BOLD signal in contralateral and in unstimulated ipsilateral regions, which depended on grouping and whether the transient was part of an attended or unattended stimulus. This pattern is consistent with a combination of localized bottom-up activation and spatially undifferentiated top-down feedback. Such global attentional effects have often been observed in studies of feature-based attention, where attention to specific features modulates brain activity across the visual field (Saenz et al., 2002; Martinez-Trujillo and Treue, 2004).

Kim and Verghese (2012) found widespread EEG correlates throughout visual cortex, while here the effects were significant only in V1. The involvement of object-based effects in such an early retinotopic area concurs with another recent fMRI study (Ciaramitaro et al., 2011), and is also consistent with another report that attentional spreading across motion and color features may depend on primary representations of spatiotemporal correspondences between these features, which could be represented at early stages of visual processing (Melcher et al., 2005). We observed similar trends in visual areas V4 and V5/MT, but these were non-significant, possibly due to insufficient statistical power. Such trends might reflect specific modulation of color and motion representations, or more general feed-forward effects of selection imposed in V1.

### Theoretical proposals

If the classical assumptions of facilitatory and automatic object-based attention cannot explain our findings, what are the alternatives? One possibility is that grouping cues can constrain not only attentional facilitation of potentially relevant stimuli, but also suppression of non-target features when they are completely irrelevant (Tipper et al., 1991; Fuentes et al., 1998). For example, when the stimuli are segmented by motion into two separate objects, relevant target features of the currently attended object can be selected, while the irrelevant transient belonging to the same object is suppressed; however this suppression may be constrained by the boundaries of the attended object and thus does not spread to the transient belonging to the ignored object, which still evokes a cortical response. Consistent with this, suppression did also affect the irrelevant hemifield in the grouping condition (e.g., compare right datapoints of Figure 6). However puzzlingly, transient stimuli in the relevant hemifield now evoked stronger responses than with ungrouped stimuli. This apparent loss of within-object feature selectivity is difficult to explain under the above account of object-based suppression alone. This discrepancy might be explained with the additional assumption (discussed earlier) that the grouping condition in our study creates a segmentation problem (analogs to that encountered with overlapping stimuli; see Wegener et al., 2008). In this case selective attention to the target on one hemifield may also be aided by nominally irrelevant transient cues, which define the context of the hemifield in which the target appears.

The observed combination of facilitatory and suppressive pattern of results is also consistent with the theory of biased competition (Desimone and Duncan, 1995) between “objects,” as defined by grouping cues and including their component parts and features (Vecera and Behrmann, 2001). According to this framework, different objects compete for representation, and top-down bias can be applied to allow one selected object to win this competition (while the competitors are simultaneously suppressed). Our results might be explained by additionally assuming that *competition is stronger between stimuli comprising a group*, than between stimuli associated with separate groups. On this assumption, the irrelevant hemifield and its transient flashes compete for attention more vigorously in the grouped condition compared to the ungrouped condition. To reduce this distraction, stronger top-down bias is needed in favor of the relevant hemifield. This hemifield bias simultaneously explains the increase in response to transients (which may help to define the relevant stimulus area, see the segmentation argument above), and the decrease in activity for transients in the irrelevant hemifield (blue lines in Figure 6). In the ungrouped case (green lines in Figure 6), there would be less competition between hemifields, so that relevant target features can be selected without much interference from the opposite hemifield. Thus less top-down bias is needed to select the relevant hemifield. However in the ungrouped condition there would still be strong competition within the attended hemifield from its irrelevant transients, compared to transients in the opposite hemifield. Top-down bias might then be needed to suppress these non-target features specifically whenever they occur within the attended hemifield. This would explain why the response to the transient appeared lower in the attended hemifield than in the unattended hemifield, in the ungrouped condition. It might be advantageous to apply this bias as early as possible in the processing stream, to achieve maximum leverage over the balance of competition between features, and hemifields, which could explain why the most robust effects of modulation were observed in primary visual cortex.

Increased within-group competition would also be consistent with two aspects of the block-related results (Figure 4): firstly, the apparent enhancement of the difference between hemispheres contralateral and ipsilateral to the attentional cue, under grouping compared to no grouping, is consistent with stronger effects of bias in the former case; and secondly, the generally lower block-related activation across visual areas for grouped vs. ungrouped stimuli, consistent with greater mutual inhibition between the hemifield representations (c.f. Kastner et al., 1998).

The proposed assumption of intensified competition within groups offers an alternative explanation of some more classical findings from object-based attention research, for example why “flanker” stimuli belonging to the same group as a target compete more vigorously for control over responding, than when displayed in a segmented context (Baylis and Driver, 1992; Zhao et al., 2013). Furthermore, enhanced competition for attention within groups could be of functional benefit by promoting rapid redeployment of attention to different locations within the same object. This could explain the lower response times observed in Posner-cuing paradigms to targets following invalid cueing to a location elsewhere within a closed contour (Egly et al., 1994), which is more commonly explained in terms of facilitatory attentional spreading. However, in contrast to an attentional mechanism based only on automatic mutual facilitation of object parts and features, this enhanced competition might also allow the perceiver to “drill down” to just one component of a group when this is uniquely relevant (e.g., the check-size targets in one hemifield), while suppressing other irrelevant component when they are fully irrelevant (e.g., the transient events, when they are truly of no use for segmenting the hemifields).

To conclude, the results of this study confirm that allocation of attentional resources, as indexed by changes in the BOLD signals in V1 evoked by task-irrelevant stimulus flashes, is strongly dependent on global cues for grouping. However a complex pattern of apparently suppressive as well as facilitatory effects reveals a wider gamut of behavior than expected by current theories of object-based attention. In contrast to many previous findings, our results suggest that allocation of attention within the bounds of an object is neither always automatic nor always facilitatory, but can be task-dependent and suppressive for truly irrelevant stimuli. Such new patterns highlight the great flexibility, rather than the limitations, of our ability to selectively ignore irrelevant information, and to drill down to the level of detail at which specifically task-relevant information may be found.

### Conflict of interest statement

The authors declare that the research was conducted in the absence of any commercial or financial relationships that could be construed as a potential conflict of interest.

## Supplementary material

Movies 1 and 2 **Movies show excerpts of typical stimulus sequences from the Grouped and Ungrouped blocked conditions, respectively**.

Supplementary Figures 1 and 2 **Results for non-normalized analysis of Block-related (Supplementary Figure 1) and Event-related (Supplementary Figure 2) results**.
