# Supplementary figures and images for: fMRI correlates of object-based attentional facilitation vs. suppression of irrelevant stimuli, dependent on global grouping and endogenous cueing
Source: Front Integr Neurosci. 2014 Feb 10;8:12. doi: 10.3389/fnint.2014.00012 (PMC3918649; doi:10.3389/fnint.2014.00012)

Supplementary Figure 1

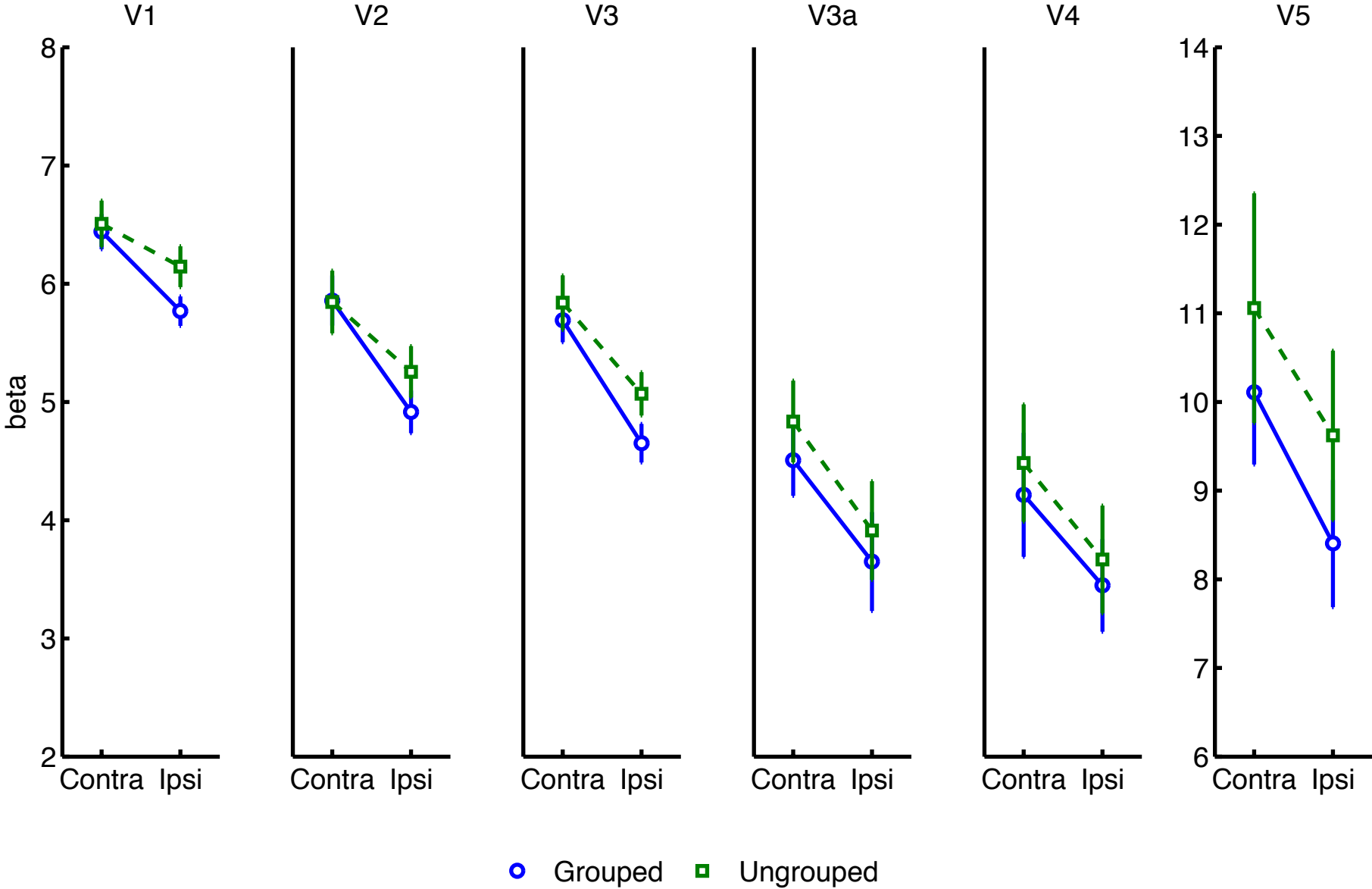

Supplementary Figure 2

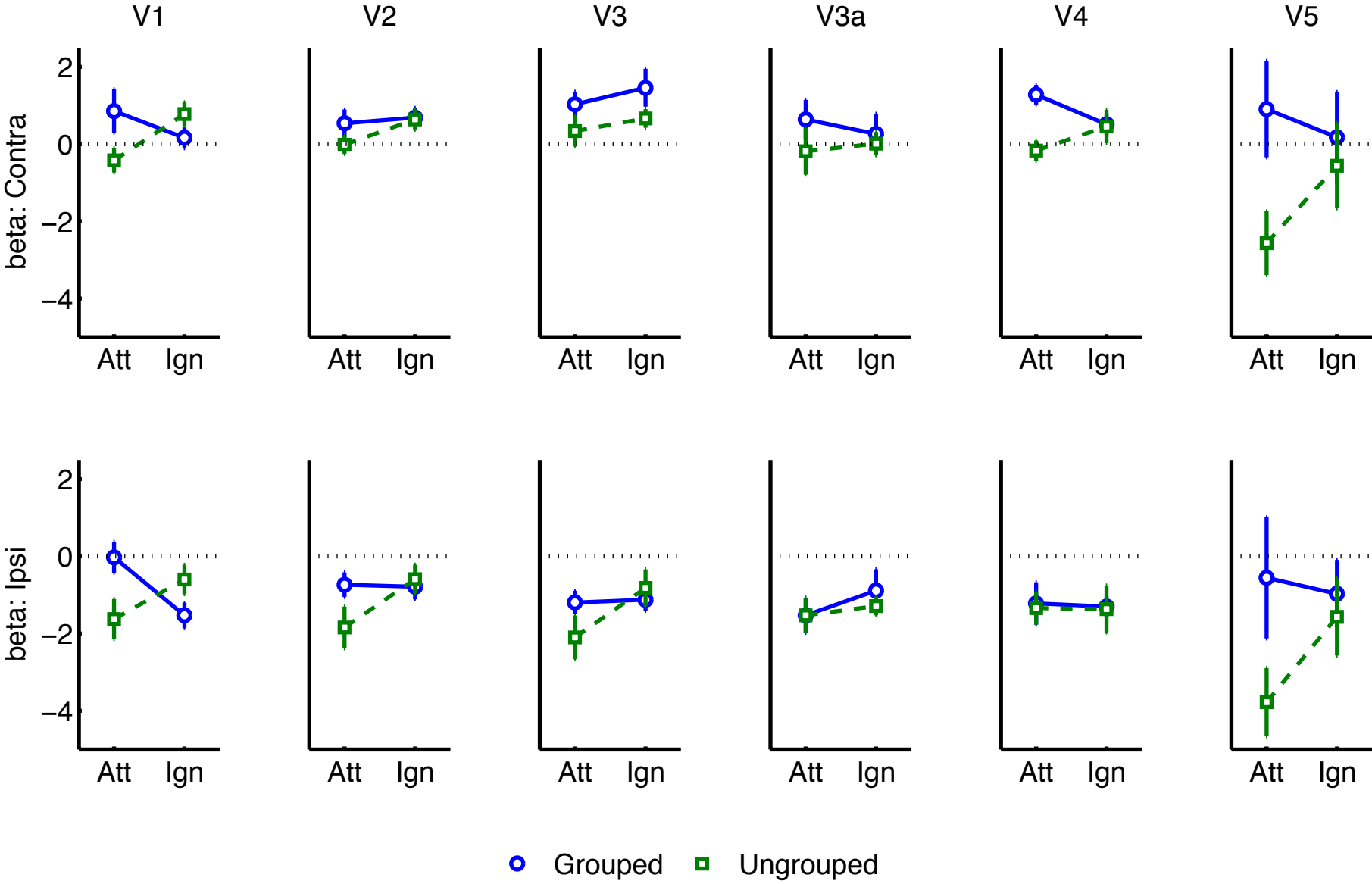

Supplement: Supplementary Figures 1 and 2 — Results for non-normalized analysis of Block-related (Supplementary Figure 1) and Event-related (Supplementary Figure 2) results. [file DataSheet1.PDF]
